# Performance comparison of gel and capillary electrophoresis-based microsatellite genotyping strategies in a population research and kinship testing framework

**DOI:** 10.1186/s13104-021-05861-9

**Published:** 2021-12-07

**Authors:** Julissa J. Sánchez-Velásquez, Lorenzo E. Reyes-Flores, Carmen Yzásiga-Barrera, Eliana Zelada-Mázmela

**Affiliations:** Laboratory of Genetics, Physiology, and Reproduction, Faculty of Sciences, Universidad Nacional del Santa, Av Universitaria s/n, 02712 Nuevo Chimbote, Peru

**Keywords:** Capillary electrophoresis, Genetic diversity, Polyacrylamide gel electrophoresis, Relatedness

## Abstract

**Objective:**

The advancement of molecular techniques in an era in which high-throughput sequencing has revolutionized biology renders old-fashioned alternatives to high-throughput methods obsolete. Such advanced molecular techniques, however, are not yet accessible to economically disadvantaged region-based laboratories that still obtain DNA profiles using gel-based techniques. To explore whether cost-efficient techniques can produce results that are as robust as those obtained using high-throughput methods, we compared the performance of polyacrylamide gel electrophoresis (PAGE)- and capillary electrophoresis (CE)-derived genomic data in estimating genetic diversity and inferring relatedness using 70 individuals of fine flounder (*Paralichthys adspersus*) selected from a hatchery population and genotyped for five microsatellite loci.

**Results:**

Here, we show that PAGE- and CE-derived genomic datasets yield comparable genetic diversity levels regarding allelic diversity measures and heterozygosity. However, relatedness inferred from each dataset showed that the categorization of dyads in the different relationship categories strongly differed. This suggests that while scientists can reliably use PAGE-derived genomic data to estimate genetic diversity, they cannot use the same for parentage testing. The findings could help laboratories committed to population research not be discouraged from using the PAGE system if high-throughput technologies are unavailable and the method is adequate to address the biological question.

**Supplementary Information:**

The online version contains supplementary material available at 10.1186/s13104-021-05861-9.

## Introduction

Ever since their discovery in 1981 [1], the usefulness of microsatellites, due to their hypervariability and ubiquitous occurrence, has been astounding to geneticists [2]. Microsatellites are short tandem repeats of almost anything from 1 to around 6 bp [2]. They are highly informative, codominant, and transferable among phylogenetically-related species [3]. As such, microsatellites are powerful tools commonly used in population research to infer genetic diversity, genetic structure, and mating systems [4–6]. Microsatellites have also found their application in linkage-disequilibrium, in which associations between markers and traits are searched, and hitchhiking mapping, in which genome-wide surveys are used to identify regions showing positive selection [7, 8]. However, to obtain reliable information, microsatellite genotyping should be performed with extreme accuracy since high error rates could inject bias into the downstream analysis and, therefore, alter ecological and evolutionary conclusions [9, 10].

Besides allelic dropouts and null alleles, the most common errors during microsatellite genotyping include contaminant DNA, incorrect data entry, and scoring errors [11]. Although most of these errors can be detected if the genotypification is repeated, the proportion of mis-scored alleles, which can be up to 80%, will depend on the researchers’ genotyping method [12]. The gold standard for microsatellite genotyping is capillary electrophoresis (CE), a technology that accurately scores the alleles owing to its technology that implements automated allele-call programs [13]. However, because this method requires sophisticated instruments, it is usually impractical for many laboratories in countries having no access to cutting-edge technologies [14, 15]. Nonetheless, more costly-effective methods such as polyacrylamide gel electrophoresis (PAGE) have been remarkably resilient in competition against sequencing techniques [16], and are still being utilized by researchers, especially in population research [17–19]; though the accuracy of their results compared to those obtained using high-throughput (HTP) methods is still arguable [12, 20–22].

Here, we present a direct comparison between PAGE and CE methods by applying them in the genotypification of five microsatellite loci in an economically important fish population. To the best of our knowledge, this study is the first of its kind that compares not only allele frequency data but also the different genetic diversity parameters analyzed in population research. Moreover, this study evaluates the performance of the PAGE method by comparing the results of relatedness analysis to those obtained using the CE method.

## Main text

### Materials and methods

#### DNA extraction and PCR amplification

Total 70 adults of *Paralichthys adspersus* (Steindachner, 1867) belonging to a hatchery population were genotyped by PAGE and CE methods using the flanking primers of five microsatellite loci developed for *P. olivaceus* (Temminck & Schlegel, 1846): *Poli9TUF*, *Poli28TUF* [23], *Po35*, *Po91* [24], and *KOP45* [25]. DNA was extracted from approximately 20 mg of caudal fin tissue using the SDS-proteinase K/phenol–chloroform digestion method adapted from Taggart et al. [26]. The PCR reactions were performed in 7 μL reaction mixture containing 4.32 μL PCR water, 0.66 μL Taq Buffer KCl-MgCl2 (10X), 0.51 μL MgCl_2_ (25 mM), 0.33 μL dNTPs (2.5 mM), 0.06 μL forward/reverse primer (12.5 μM each), 0.06 μL Maximo Taq DNA Polymerase (5 U/μL) (GeneON, Deutschland, Germany), and 1 μL DNA (25 ng/μL). Thermal cycling conditions for each locus are detailed in Additional file 1: Table S1.

#### PAGE

PCR amplification products were visualized on 12% polyacrylamide gels following the standard Laemmli system for discontinuous gel electrophoresis [27]. Gels containing 12% separating gel and 4% stacking gel were prepared from a stock solution of 40% Acrylamide/Bis Solution 19:1 (Bio-Rad, California, USA). The separating gel contained 2667 µL distilled water, 1800 µL of 40% Acrylamide/Bis solution, and 1500 µL Tris–HCl (1.5 M, pH 8.8). 7 cm separating gels were prepared into a plate sandwich of a total length of 10 cm and with a 1-mm-thick spacer. The gels were polymerized chemically by the addition of 3 µL tetramethylethylenediamine (TEMED) and 30 µL ammonium persulfate (APS) (10%). The stacking gel contained 1906.38 µL distilled water, 300 µL of 40% Acrylamide/Bis solution, and 760.62 µL Tris–HCl (0.5 M, pH 6.8), and was polymerized in the same way as for separating gel. After gel polymerization, the plate sandwich was placed into the Ommi PAGE CVS10D gel system (Cleaver Scientific Ltd., Warwickshire, UK). Samples were prepared using 6 µL of PCR amplification product and 1 µL of 6X DNA loading dye (Thermo Scientific, Massachusetts, USA). Electrophoresis was carried out with a voltage of 85 V for about 2.5 h. DNA bands were revealed using a silver nitrate staining adapted from Rangel-Villalobos and colleagues [28]. Allele sizes were estimated in comparison with a 300 bp ladder (Thermo Scientific) using Quantity One^®^ 1-D Analysis software (Bio-Rad).

#### CE

PCR amplification products were also genotyped by means of automated parallel CE using the Fragment Analyzer™ Automated CE System (Agilent, California, USA). Separation gel and samples were prepared using the DNF-900 dsDNA Reagent Kit (Agilent) following the manufacturer’s protocol. Allele scoring was performed using PROSize data analysis software (Agilent) by interpolating their position to a 35–500 bp DNA marker and 75–400 bp range DNA ladder (Agilent).

#### Genetic diversity

Typographic errors, i.e., the misinterpretation of allele banding patterns because of stutter bands, and allelic dropout were determined using Micro-Checker v.2.2.3 [29]. The frequency of null alleles was calculated using ML-Null Freq v.1.0 [30]. The polymorphic information content (PIC) at each locus was calculated using CERVUS v.3.0.7 [31]. The diversity for each locus was quantified as the number of alleles per locus (A), the effective number of alleles (a_e_), allelic frequency, observed heterozygosity (Ho), and expected heterozygosity (He) using GenAlEx v.6.5 [32]. Allelic richness (R) was evaluated using HP-Rare v.1.0 [33]. Statistical differences between the genetic diversity measures obtained from the PAGE- and CE-derived genomic datasets were subsequently tested using unpaired Student’s t tests on GraphPad Prism v.7.0. The obtained *P* values  < 0.05 were considered to be statistically significant. Further data visualization was performed in R [34].

#### Relatedness estimation

The coefficient of relatedness (*r*_*xy*_) was estimated using the method-of-moment estimator developed by Wang (*r*_*w*_) [35] through Coancestry v.1.2.1 [36]. The cutoff value to group the formed dyads in the different relationship categories, full-sibs (FS), half-sibs (HS), and unrelated (UR), was established as the midpoint between the arithmetic mean *r*_*w*_ values of any two adjacent distributions, as suggested by Blouin et al. [37, 38]. Following Blouin et al. suggestion, FH would be the dyads with *r*_*w*_ values between 0.5 and 0.375, HF the dyads with *r*_*w*_ values between 0.375 and 0.125, and UR the dyads with an *r*_*w*_ lower than 0.125. It has to be noted, however, that although the *r*_*w*_ was developed from the identical by descent (IBD)‐based concept of relatedness, where *r*_*xy*_ can only go from 0 to 1 if neither of the two individuals being compared is inbred, this method-of-moment estimator provide negative values if the average relatedness among sampled individuals becomes close to zero [39, 40]. This occurs because to calculate *r*_*xy*_, the allele frequency data is estimated from the current sample instead of an ancestral population (as assumed when the estimators were developed) [39]. Negative values, however, have biological meaning if they are understood as the correlation of homologous genes between and within individuals due to shared ancestry as conceived by Wright [39, 41] in the original correlation concept of relatedness. Thus, if negative values are found, they indicate the individuals being compared are less related in ancestry than the average [39]; they belong to the UR category.

### Results

#### Genetic diversity

**Fig. 1:**
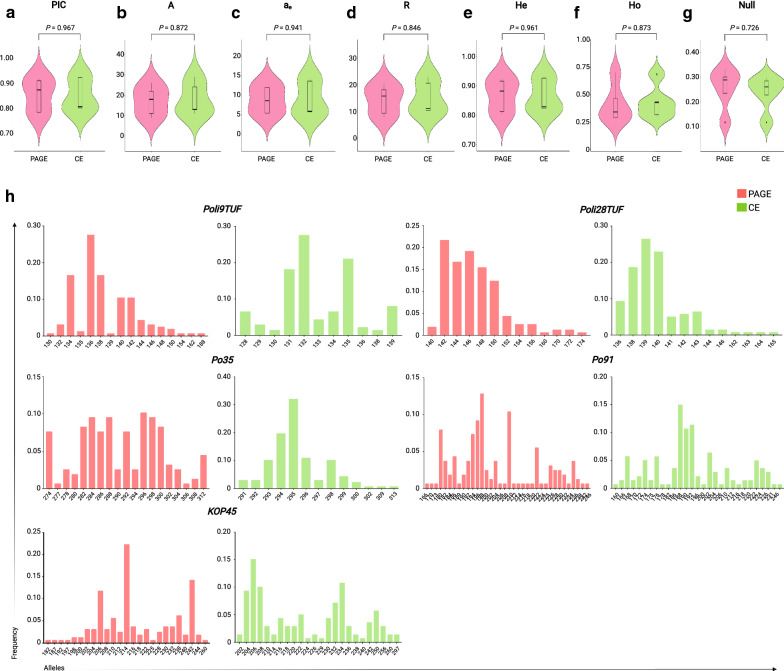
Polyacrylamide gel electrophoresis (PAGE)- and capillary electrophoresis (CE)-derived genomic data give comparable genetic diversity measures. Total 70 samples of a captive population of fine flounder (*Paralichthys adspersus*) were genotyped for five microsatellite loci using PAGE and CE methods, and the genetic diversity levels obtained from both genomic datasets were compared. **a-f** Violin plots with included boxplots summarizing the data distribution of different genetic diversity parameters. **g** Violin plots with included boxplots summarizing the distribution of null allele frequencies. Each box plot shows the median (middle line) and interquartile range (boxes). The bottom and top of each box indicate the 25th and 75th percentiles, while whiskers represent the minimum and maximum. **h** Allele frequency spectra of the five microsatellite loci used. Statistical analysis was performed using unpaired t tests. *PIC* polymorphic information content; *A* number of alleles; *a*_*e*_ effective number of alleles; *R* allelic richness; *Ho* observed heterozygosity; *He* expected heterozygosity

Except for *Poli9TUF*, the number of alleles detected by PAGE and CE methods varied depending on the specific locus (Additional file 1: Table S2). Particularly in eight samples, the CE method was more sensitive and able to separate PCR products that differed by only 2 bp. The total number of alleles in both derived datasets ranged from 86 to 90. The genetic diversity estimated from both genomic datasets was also not significantly different (in all cases *P*  > 0.05) (Fig. 1a–f). The PAGE-derived genomic data gave a mean PIC value of 0.85 ± 0.008. The average A, a_e_, and R were evaluated as 17.20 ± 0.859, 8.77 ± 0.431, and 14.03 ± 0.661. The average value of He was 0.87 ± 0.007, while that of Ho was 0.42 ± 0.021. Similarly, using CE-derived genomic data, we inferred the mean PIC value as 0.85 ± 0.008. The average A, a_e_, and R were 18.00 ± 0.956, 8.97 ± 0.532, and 16.13 ± 0.739. The average value of He was 0.87 ± 0.007, while that of Ho was 0.43 ± 0.018. The frequency of null alleles obtained from both datasets also showed no significant differences (*P*  = 0.726) (Fig. 1g). The allele frequency spectra obtained by both methods were also comparable, except for *Po35* and *KOP45* (Fig. 1h).

#### Patterns of relatedness

**Fig. 2:**
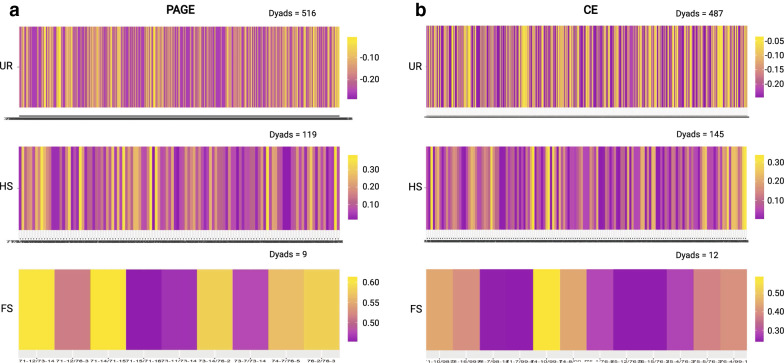
Differences in relatedness estimation between polyacrylamide gel electrophoresis (PAGE)- and capillary electrophoresis (CE)-derived genomic datasets. Total 70 samples of a captive population of fine flounder (*Paralichthys adspersus*) were genotyped for five microsatellite loci using PAGE and CE methods, and relatedness among males and females was inferred using the Wang estimator (*r*_*w*_). **a** Heat maps showing the *r*_*w*_ values calculated from the PAGE-derived genomic dataset for all dyads within each relationship category. **b** Heat maps showing the *r*_*w*_ values calculated from the CE-derived genomic dataset for all dyads within each relationship category. Heat maps were made to compare and visualize the difference in *r*_*w*_ values and the number of dyads obtained from the PAGE- and CE-derived genomic datasets. Each bar in heat maps represents one dyad, and colors show the variation in *r*_*w*_ values. *UR* unrelated; *HS* half-sibs; *FS* full-sibs

The average values for the *r*_*w*_ estimator inferred from the PAGE and CE datasets supported the category unrelated as the most probable type of relationship between the individuals from the captive population of *P. adspersus*, with average values ranging from − 0.111 to − 0.093. However, strong differences in the categorization of HS and FS were observed (Fig. 2a, b). For HS, only 22.76% of the dyads identified as such using the CE-derived dataset coincided with the dyads identified as HS using the PAGE dataset, while both datasets shared no dyad in the FS category. Instead, out of the total dyads identified as FS using the CE-derived dataset, we categorized 36.36% as HS and 63.64% as UR using the PAGE-derived dataset.

To validate our analyses, we also determined genetic diversity and relatedness from 40 wild individuals of *P. adspersus* by using the PAGE and CE genotyping methods. The results were similar to those obtained using the captive population: no difference in genetic diversity but great differences in kinship assignment were found (Additional file 1: Figs. S1, S2). Therefore, while microsatellite allelic data obtained from the PAGE system seem reliable to determine genetic diversity, the same seems unreliable to infer relatedness compared to that obtained using the CE method.

## Discussion

Increasing the studies that can face the ongoing biodiversity and climate crisis in countries without access to cutting-edge technologies will need that the relevance of their studies be valued for their biological significance rather than the fanciness of the technique utilized [15]. However, the analytical and methodological limitations of the allegedly old-fashioned methods and their adequateness to address the biological question asked need to be considered. In this study, we demonstrated that the PAGE- and CE-derived genomic data yield nearly identical genetic diversity levels for a captive population of *P. adspersus*. Compared to the CE method, however, the PAGE method failed to provide similar allele frequency spectra in two out of the five microsatellite loci used, a phenomenon mainly attributed to the misinterpretation of allele banding patterns [12, 20]. Since two previous studies with the PAGE method have provided allele frequency data that agree with that obtained using the CE method, by as much as 99% [16, 21], methodological issues in this study can also not be ruled out as an error source. These findings highlight the limitations of non-HTP methods for accurate allele scoring and present avenues for improved error reduction by adjusting the data collection process. For example, Pagel et al. [21] indicate that to increase the power of resolution and avoid doubtful alleles, the PAGE’s electrophoretic conditions can be improved by decreasing the voltage and increasing the gel concentration and running time, conditions that even though make the protocols more time consuming and labor-intensive, ultimately allow the visualization of one-base differences.

Although the discrepancies in allele frequency between PAGE- and CE-derived genomic datasets did not affect genetic diversity estimation, it is considerable enough to bear in mind that they did affect more complex analyses such as kinship assignation, in which a single erroneously identified allele leads to misinterpretations [20, 21]. Indeed, we obtained different patterns of relatedness while inferring relatedness from each dataset. Many studies have shown that when the genomic data are teeming with mistyping errors, the actual relatedness among individuals is difficult to detect, as error rates as low as 0.01 per allele can result in a rate of false paternity exclusion exceeding 20% [9, 11, 12]. An alternative explanation for the disagreement between both datasets in terms of relatedness is the presence of low heterozygosity in the captive population (less than 50% on average), which can prevent precise parentage assignation unless a large number of microsatellite loci are combined with a near-complete sampling of the parental generation [42]. If the analysis of more loci through the PAGE method can lead to the correct categorization of dyads, however, requires further investigation. Nevertheless, as parentage testing is a powerful tool to study life history and establish sustainable breeding programs if estimated accurately [42], our results showed that researchers should not rely on PAGE-derived genomic data to infer relatedness; instead, they should utilize genomic data generated from HTP methods, which can accomplish allele sizing with higher resolution and greater accuracy [20].

## Conclusion

Typographical errors within the PAGE-derived genomic data led to discrepancies in relatedness patterns; nonetheless, the PAGE-and CE-derived genomic datasets gave the same conclusions regarding genetic diversity. Therefore, such findings suggest that the PAGE system is an efficient and less costly alternative to the HTP methods in a population research framework, with the advantage that any laboratory with minimum infrastructure can accomplish it.

## Limitations

While the evidence we have shown provides strong support for the effect of the genotyping technique in kinship assignment, our study is not without certain caveats. To address some of them, we should mention that the accuracy of relatedness estimation improves as the number of loci increases [43]. For instance, while the proportion of successfully identified parent–offspring dyads can be up to 0.75 if five microsatellite loci are used, this proportion increases to 0.95 if 20 microsatellites are used [43]. Moreover, it has been shown that genotyping with less than 17 microsatellite loci leads to significant deviations in population genetic results by affecting the stability of genetic distance parameters [3, 44, 45]. Because an increasing number of microsatellite loci could have affected the results of both PAGE- and CE-derived genomic datasets, the assessment of this variable together with the technique used warrants further investigation. Finally, this study considers relatedness analysis based on the empirical kinship coefficient alone estimated using genomic data since our dataset did not include pedigree information. Therefore, further study on the precision of the CE method in estimating true relatedness is needed.

## Supplementary Information

**Additional file 1: Figure S1. **Capillary electrophoresis (CE)- and polyacrylamide gel electrophoresis (PAGE)-derived genomic datasets give comparable genetic diversity measures. Total 40 samples of a wild population of fine flounder (*Paralichthys adspersus*) were genotyped for five microsatellite loci using CE and PAGE methods, and the genetic diversity levels obtained from both genomic datasets were compared. **a-f** Violin plots with included boxplots summarizing the data distribution of different genetic diversity parameters. **g** Violin plots with included boxplots summarizing the distribution of null allele frequencies. Each box plot shows the median (middle line) and interquartile range (boxes). The bottom and top of each box indicate the 25th and 75th percentiles, while whiskers represent the minimum and maximum. **h** Allele frequency spectra of the five microsatellite loci used. Statistical analysis was performed using unpaired t tests. PIC, polymorphic information content; *A* number of alleles; *a*_*e*_ effective number of alleles; *R* allelic richness; *Ho* observed heterozygosity; *He* expected heterozygosity. **Figure S2. **Differences in relatedness estimation between capillary electrophoresis (CE)- and polyacrylamide gel electrophoresis (PAGE)-derived genomic datasets. Total 40 samples of a wild population of fine flounder (*Paralichthys adspersus*) were genotyped for five microsatellite loci using CE and PAGE methods, and relatedness among males and females was inferred using the Wang estimator (*r*_*w*_). **a** Heat maps showing the *r*_*w*_ values calculated from the CE-derived genomic dataset for all dyads within each relationship category. **b** Heat maps showing the *r*_*w*_ values calculated from the PAGE-derived genomic dataset for all dyads within each relationship category. Heat maps were made to compare and visualize the difference in *r*_*w*_ values and the number of dyads obtained from the CE- and PAGE-derived genomic datasets. Each bar in heat maps represents one dyad, and colors show the variation in *r*_*w*_ values. *UR* unrelated; *HS* half-sibs; *FS* full-sibs. **Table S1.** PCR conditions and size range of the fragments for each microsatellite locus. **Table S2.** Genetic diversity obtained from capillary electrophoresis (CE)- and polyacrylamide gel electrophoresis (PAGE)-derived genomic data for a captive population of *Paralichthys adspersus*. Repeat motif, annealing temperature (Ta), frequency of null alleles, polymorphic information content (PIC), number of alleles per locus (A), effective number of alleles (a_e_), allelic richness (R), and observed and expected heterozygosity (Ho/He) for each microsatellite locus are shown. **Table S3.** Genetic diversity obtained from capillary electrophoresis (CE)- and polyacrylamide gel electrophoresis (PAGE)-derived genomic data for a wild population of *Paralichthys adspersus*. Repeat motif, annealing temperature (Ta), frequency of null alleles, polymorphic information content (PIC), number of alleles per locus (A), effective number of alleles (a_e_), allelic richness (R), and observed and expected heterozygosity (Ho/He) for each microsatellite locus are shown.

## Data Availability

The datasets used and/or analyzed during the current study are available from the corresponding author on reasonable request.

## Abbreviations

A: Number of alleles
a_e_: Effective number of alleles
APS: Ammonium persulfate
CE: Capillary electrophoresis
FS: Full-sibs
He: Expected heterozygosity
Ho: Observed heterozygosity
HS: Half-sibs
PAGE: Polyacrylamide gel electrophoresis
PIC: Polymorphic information content
R: Allelic richness
*r*_*w*_: Wang estimator
*r*_*xy*_: Coefficient of relatedness
TEMED: Tetramethylethylenediamine
UR: Unrelated
Allele: One of the possible forms of a gene at a given locus
Allelic dropout: Source of missing data in which one or both allelic copies at a locus fail to be amplified by PCR
Allele frequency: Relative proportion of an allele within a population
Allelic richness: Number of alleles in a set of populations determined taking into account the smallest population size
Genetic relatedness: The probability that two individuals share an allele due to recent common ancestry
Heterozygosity: The proportion of individuals within a population that carry two different alleles at a locus
Microsatellite hypervariability: Accumulation of length mutations during replication at a given microsatellite locus
Null alleles: Amplification failure due to mutations in the binding site of at least one primer
PIC: Discriminatory power of loci considering allele number and relative frequency
Stuttering: Series of minor peaks immediately adjacent to the major peak for a given locus in a gel
