# Supplementary material for: Performance comparison of gel and capillary electrophoresis-based microsatellite genotyping strategies in a population research and kinship testing framework
Source: BMC Res Notes. 2021 Dec 7;14:444. doi: 10.1186/s13104-021-05861-9 (PMC8650532; doi:10.1186/s13104-021-05861-9)
Supplement: Supplementary file 1 — Additional file 1: Figure S1. Capillary electrophoresis (CE)- and polyacrylamide gel electrophoresis (PAGE)-derived genomic datasets give comparable genetic diversity measures. Total 40 samples of a wild population of fine flounder (Paralichthys adspersus) were genotyped for five microsatellite loci using CE and PAGE methods, and the genetic diversity levels obtained from both genomic datasets were compared. a-f Violin plots with included boxplots summarizing the data distribution of different genetic diversity parameters. g Violin plots with included boxplots summarizing the distribution of null allele frequencies. Each box plot shows the median (middle line) and interquartile range (boxes). The bottom and top of each box indicate the 25th and 75th percentiles, while whiskers represent the minimum and maximum. h Allele frequency spectra of the five microsatellite loci used. Statistical analysis was performed using unpaired t tests. PIC, polymorphic information content; A number of alleles; ae effective number of alleles; R allelic richness; Ho observed heterozygosity; He expected heterozygosity. Figure S2. Differences in relatedness estimation between capillary electrophoresis (CE)- and polyacrylamide gel electrophoresis (PAGE)-derived genomic datasets. Total 40 samples of a wild population of fine flounder (Paralichthys adspersus) were genotyped for five microsatellite loci using CE and PAGE methods, and relatedness among males and females was inferred using the Wang estimator (rw). a Heat maps showing the rw values calculated from the CE-derived genomic dataset for all dyads within each relationship category. b Heat maps showing the rw values calculated from the PAGE-derived genomic dataset for all dyads within each relationship category. Heat maps were made to compare and visualize the difference in rw values and the number of dyads obtained from the CE- and PAGE-derived genomic datasets. Each bar in heat maps represents one dyad, and colors show the variation i [file 13104_2021_5861_MOESM1_ESM.docx]

**Supplementary information**





**Fig. S1. Capillary electrophoresis (CE)- and polyacrylamide gel electrophoresis (PAGE)-derived genomic datasets give comparable genetic diversity measures.** Total 40 samples of a wild population of fine flounder (*Paralichthys adspersus*) were genotyped for five microsatellite loci using CE and PAGE methods, and the genetic diversity levels obtained from both genomic datasets were compared. **a-f.** Violin plots with included boxplots summarizing the data distribution of different genetic diversity parameters. **g.** Violin plots with included boxplots summarizing the distribution of null allele frequencies. Each box plot shows the median (middle line) and interquartile range (boxes). The bottom and top of each box indicate the 25th and 75th percentiles, while whiskers represent the minimum and maximum. **h.** Allele frequency spectra of the five microsatellite loci used. Statistical analysis was performed using unpaired t-tests. PIC, polymorphic information content; A, number of alleles; a_e_, effective number of alleles; R, allelic richness; Ho, observed heterozygosity; He, expected heterozygosity.


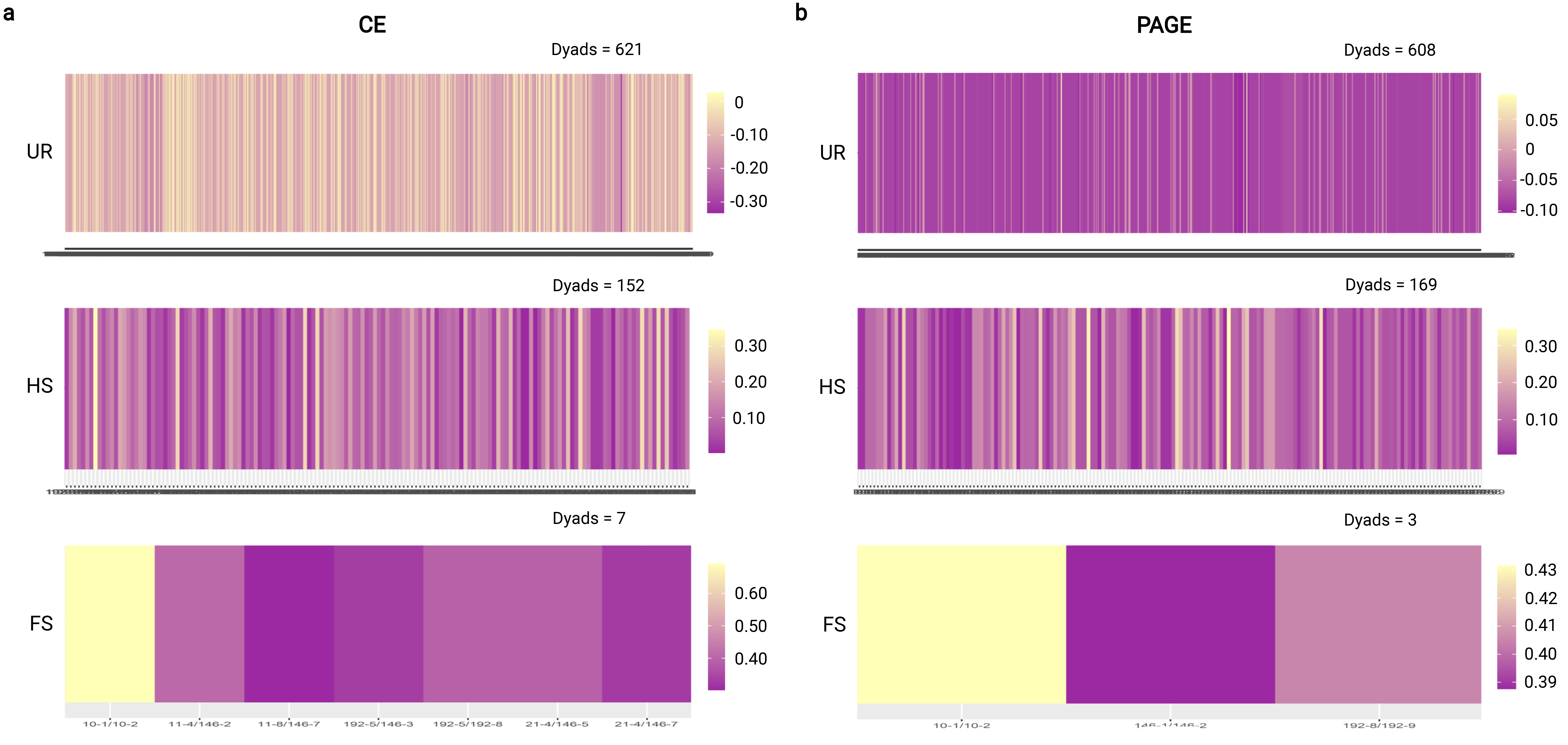


**Fig. S2. Differences in relatedness estimation between capillary electrophoresis (CE)- and polyacrylamide gel electrophoresis (PAGE)-derived genomic datasets.** Total 40 samples of a wild population of fine flounder (*Paralichthys adspersus*) were genotyped for five microsatellite loci using CE and PAGE methods, and relatedness among males and females was inferred using the Wang estimator ($r_{W}$). **a.** Heat maps showing the $r_{W}$values calculated from the CE-derived genomic dataset for all dyads within each relationship category. **b.** Heat maps showing the $r_{W}$values calculated from the PAGE-derived genomic dataset for all dyads within each relationship category. Heat maps were made to compare and visualize the difference in $r_{W}$values and the number of dyads obtained from the CE- and PAGE-derived genomic datasets. Each bar in heat maps represents one dyad, and colors show the variation in $r_{W}$values. UR, unrelated; HS, half-sibs; FS, full-sibs.

**Table S1. PCR conditions and size range of the fragments for each microsatellite locus.**

| **Locus** | **PCR conditions** | **PCR size range (bp)** |
| --- | --- | --- |
| *Poli9TUF* | 95 °C for 3 min; 28 cycles of 95 °C (30 s), 55 °C (20 s), 72 °C (12 s); and 72 °C (3 min) | 126-170 |
| *Poli28TUF* | 95 °C for 3 min; 30 cycles of 95 °C (30 s), 60 °C (30 s), 72 °C (12 s); and 72 °C (7 min) | 135-175 |
| *Po35* | 95 °C for 5 min; 28 cycles of 95 °C (25 s), 59 °C (20 s), 72 °C (28 s); and 72 °C (7 min) | 291-353 |
| *Po91* | 95 °C for 5 min; 28 cycles of 95 °C (30 s), 55 °C (20 s), 72 °C (12 s); and 72 °C (3 min) | 160-226 |
| *KOP45* | 95 °C for 3 min; 29 cycles of 95 °C (30 s), 60 °C (20 s), 72 °C (12 s); and 72 °C (3 min) | 202-260 |

**Table S2.** **Genetic diversity obtained from capillary electrophoresis** **(CE)- and polyacrylamide gel electrophoresis (PAGE)-derived genomic data for a captive population of *Paralichthys adspersus*.** Repeat motif, annealing temperature (Ta), frequency of null alleles, polymorphic information content (PIC), number of alleles per locus (A), effective number of alleles (a_e_), allelic richness (R), and observed and expected heterozygosity (Ho/He) for each microsatellite locus are shown.

| **Locus** |  | **Ta (°C)** | **Parameter** | **CE** | **PAGE** |
| --- | --- | --- | --- | --- | --- |
| *Poli9TUF* | (CA) | 55 | Null Freq. | 0.295 | 0.288 |
|  |  |  | PIC | 0.81 | 0.78 |
|  |  |  | A (a_e_) | 11 (5.84) | 11 (5.14) |
|  |  |  | R | 10.33 | 9.41 |
|  |  |  | Ho/He | 0.32/0.83 | 0.29/0.81 |
| *Poli28TUF* | (CA) | 60 | Null Freq. | 0.284 | 0.301 |
|  |  |  | PIC | 0.80 | 0.79 |
|  |  |  | A (a_e_) | 13 (5.69) | 9 (5.34) |
|  |  |  | R | 10.49 | 7.91 |
|  |  |  | Ho/He | 0.30/0.82 | 0.29/0.81 |
| *Po35* | (CA) | 59 | Null Freq. | 0.228 | 0.333 |
|  |  |  | PIC | 0.80 | 0.91 |
|  |  |  | A (a_e_) | 13 (5.64) | 18 (11.89) |
|  |  |  | R | 11.22 | 15.88 |
|  |  |  | Ho/He | 0.44/0.82 | 0.34/0.92 |
| *Po91* | (CA) | 55 | Null Freq. | 0.117 | 0.117 |
|  |  |  | PIC | 0.92 | 0.92 |
|  |  |  | A (a_e_) | 29 (14.14) | 26 (12.93) |
|  |  |  | R | 23.04 | 20.62 |
|  |  |  | Ho/He | 0.69/0.93 | 0.70/0.92 |
| *KOP45* | (AC) | 60 | Null Freq. | 0.260 | 0.235 |
|  |  |  | PIC | 0.92 | 0.87 |
|  |  |  | A (a_e_) | 24 (13.54) | 22 (8.56) |
|  |  |  | R | 20.64 | 18.19 |
|  |  |  | Ho/He | 0.43/0.93 | 0.46/0.88 |

**Table S3.** **Genetic diversity obtained from capillary electrophoresis** **(CE)- and polyacrylamide gel electrophoresis (PAGE)-derived genomic data for a wild population of *Paralichthys adspersus*.** Repeat motif, annealing temperature (Ta), frequency of null alleles, polymorphic information content (PIC), number of alleles per locus (A), effective number of alleles (a_e_), allelic richness (R), and observed and expected heterozygosity (Ho/He) for each microsatellite locus are shown.

| **Locus** |  | **Ta (°C)** | **Parameter** | **CE** | **PAGE** |
| --- | --- | --- | --- | --- | --- |
| *Poli9TUF* | (CA) | 55 | Null Freq. | 0.301 | 0.346 |
|  |  |  | PIC | 0.84 | 0.89 |
|  |  |  | A (a_e_) | 14 (7.06) | 19 (10.45) |
|  |  |  | R | 11.19 | 15.08 |
|  |  |  | Ho/He | 0.30/0.86 | 0.28/0.90 |
| *Poli28TUF* | (CA) | 60 | Null Freq. | 0.212 | 0.263 |
|  |  |  | PIC | 0.86 | 0.93 |
|  |  |  | A (a_e_) | 18 (7.88) | 29 (14.68) |
|  |  |  | R | 13.59 | 20.60 |
|  |  |  | Ho/He | 0.48/0.87 | 0.42/0.93 |
| *Po35* | (CA) | 59 | Null Freq. | 0.376 | 0.382 |
|  |  |  | PIC | 0.80 | 0.94 |
|  |  |  | A (a_e_) | 11 (5.62) | 24 (16.24) |
|  |  |  | R | 9.62 | 19.50 |
|  |  |  | Ho/He | 0.21/0.82 | 0.20/0.94 |
| *Po91* | (CA) | 55 | Null Freq. | 0.157 | 0.093 |
|  |  |  | PIC | 0.99 | 0.96 |
|  |  |  | A (a_e_) | 35 (23.05) | 37 (27.66) |
|  |  |  | R | 24.47 | 26.39 |
|  |  |  | Ho/He | 0.69/0.96 | 0.79/0.96 |
| *KOP45* | (AC) | 60 | Null Freq. | 0.141 | 0.119 |
|  |  |  | PIC | 0.95 | 0.96 |
|  |  |  | A (a_e_) | 30 (20.55) | 36 (24.06) |
|  |  |  | R | 22.56 | 24.72 |
|  |  |  | Ho/He | 0.72/0.95 | 0.72/0.96 |
